# Effects of Chemokine Ligand 2 on Budding of Bovine Foamy Virus

**DOI:** 10.3390/v15091867

**Published:** 2023-09-01

**Authors:** Rui Li, Zhaohuan Wang, Chenxi Liu, Wentao Qiao, Juan Tan

**Affiliations:** Key Laboratory of Molecular Microbiology and Technology, Ministry of Education, College of Life Sciences, Nankai University, Tianjin 300071, China; 1120220764@mail.nankai.edu.cn (R.L.); 1120190482@mail.nankai.edu.cn (Z.W.); 2120201045@mail.nankai.edu.cn (C.L.); wentaoqiao@nankai.edu.cn (W.Q.)

**Keywords:** bovine foamy virus, CCL2, Alix, viral budding

## Abstract

The endosomal sorting complex required for transport (ESCRT) machinery is essential for the budding of retroviruses such as human immunodeficiency virus (HIV) and bovine foamy virus (BFV), which rely on their late domain to recruit ESCRT complexes to facilitate budding. However, the impact of intracellular host proteins on BFV budding remains poorly understood. In this study, we aimed to investigate the impact of CCL2 on BFV budding and interactions with key host proteins. Our results indicate that CCL2 promotes BFV budding in an ALG-2-interacting protein X (Alix)-dependent manner by enhancing the interaction between Alix and BFV Gag (BGag). Notably, we found a link between Alix, BGag and CCL2, with Alix mediating the interaction between the latter two. Furthermore, we observed that natural host bovine CCL2 also has a facilitating role in the budding process of BFV, similar to human CCL2. Taken together, these results demonstrate that CCL2 promotes BFV budding by enhancing the Alix-BGag association.

## 1. Introduction

Foamy viruses (FVs), also known as spumaretroviruses, are a distinct genus in the Spumaretrovirinae subfamily of the Retroviridae family due to their unique replication strategies [1,2]. Similar to other retroviruses, FVs encode three structural proteins, including group-specific antigen (Gag), polymerase (Pol), and envelope (Env) glycoprotein, to form viral particles [3]. As opposed to orthoretrovirus Gag, FV Gag is not processed into matrix (MA), capsid (CA), and nucleocapsid (NC)—only a 3 kDa C-terminal peptide (p3Gag) is clipped off from the precursor instead. In the case of BFV, four Gag cleavage forms (p71, p68, p33, and p29) are observed [4]. It is worth noting that a strain of BFV (an in vitro selected cell-free infectious BFV3026 clone) screened by our laboratory is used in this paper, and that BFV Gag exists in the form of p59Gag and p56Gag [5]. Among structural proteins, Gag plays a critical role in FV budding [6], but unlike most retroviruses, FV Gag is not N-terminally myristoylated and does not have a membrane targeting function to produce cell-free Gag-only virus-like particles (VLPs) [7,8]. Instead, FV is released through the interaction of Gag with Env to form subviral particles (SVPs) [9]. Recent studies have shown that artificially fusing the N-terminal of the FV Gag proteins with myristoylation from Src, Fyn, or Lck confers a membrane targeting function for Gag, enabling FV Gag release independent of the expression of the cognate Env [10,11,12]. FVs infect various animal species, including equines, bovines, felines, and non-human primates without causing any disease [4,13,14,15]. However, as a member of the understudied non-human FVs, bovine foamy virus (BFV) can be detected in beef and raw milk, which increases the risk of human-to-human transmission [16].

Budding of enveloped viruses from cell or organelle membranes is a crucial step in the virus life cycle, often facilitated by cytokines. Retroviruses, including human immunodeficiency virus type 1 (HIV-1), prototype foamy virus (PFV), and BFV, recruit endosomal sorting complex required for transport (ESCRT) components through late domain (L domain) on their capsid precursor protein to complete budding [17,18,19]. The C-terminus of HIV-1 Gag contains two typical L domain sequences, PTAP motif and LYPX motif, which recruit tumor susceptibility 101 (Tsg101, a member of the ESCRT-I complex) and ALG-2-interacting protein X (AIP-1/Alix, an accessory protein of the ESCRT-Ⅲ complex), respectively [20,21,22]. Similarly, BFV uses two atypical L domain sequences on Gag, PLPI motif and YGPL motif, which recruit Tsg101 and Alix, respectively [19]. These proteins physically link the L domain motifs of retrovirus Gag to ESCRT-Ⅰ and ESCRT-Ⅲ complexes and are crucial for membrane fission steps like cytoplasmic division and virus budding [17]. During cytoplasmic division, the ESCRT-Ⅱ complex is recruited after the ESCRT-Ⅰ complex is loaded, followed by the recruitment of the ESCRT-Ⅲ complex, which eventually leads to membrane fission [23].

A number of cellular factors linked to the ESCRT system have been shown to play roles in retroviruses’ budding. Among these factors, Alix is the most studied. Other cytokines related to Alix are also proven to be critical for retroviral budding, such as galectin-3 [24] and chemokine ligand 2 (CCL2) [25]. CCL2, also known as monocyte chemoattractant protein-1 (MCP-1), is a member of the C-C chemokine family. Human CCL2 (hCCL2) is divided into precursors and mature bodies. The precursor molecule consists of a hydrophobic amino terminal signal peptide of 23 amino acids, which contain 99 amino acids. The mature protein contains 76 amino acids after the cleavage of signal protein [26,27]. CCL2 is constitutively expressed in most cells and primarily localized in the cytoplasm [28].

As a potent β-chemokine, CCL2 binds to its receptor CCR2 thus activating the signaling pathways which regulate the migration and infiltration of monocytes and T lymphocytes at the site of injury and infection [29]. To date, CCL2 has been shown to affect the replication of viruses, such as HIV-1 [30] and human cytomegalovirus (HCMV) [31]. CCL2 can be exploited by HIV-1 or HCMV to enhance viral replication by recruiting target cells. CCL2 is also able to affect the release of HIV-1 in MDM cells [32]. In addition, the virus also has certain effects on endogenous CCL2. CCL2 mRNA is elevated upon HIV-1 [33,34] and HCMV infection [31,35]. Although BFV recruits the ESCRT components for its own budding in a manner similar to HIV-1, no data have been published on the involvement of CCL2 in BFV budding. In this way, we report that CCL2 is capable of promoting BFV budding in an Alix-dependent manner. Moreover, we found that CCL2 enhances the interaction between BGag and Alix, furthering increase virus release.

## 2. Materials and Methods

### 2.1. Cell Culture and Transfection

Three cell lines were used in this research: HEK293T (a human kidney cell line, JLC-E2306), HeLa (human cervical cancer cells, JLC-E2224), and MDBK (bovine kidney cells, JLC-E1354). They were cultured in Dulbecco’s modified Eagle’s medium (Gibco, Thermo Fisher Scientific, Waltham, MA, USA) supplemented with 10% fetal bovine serum (FBS) (Gibco, Thermo Fisher Scientific, Waltham, MA, USA) and 1% streptomycin/penicillin (Gibco, Grand Island, NY, USA). All the cells were cultured at 37 °C with 5% CO_2_.

For transfection, cells were seeded at 70–80% confluence in either 6-well plates, 12-well plates, or 10 cm dishes. After 24 h, the required plasmids were transfected using the polyethyleneimine (PEI, Polysciences, Warrington, PA, USA) following the manufacturer’s instructions [36].

### 2.2. Plasmids and Antibodies

Human CCL2 and bovine CCL2 cDNAs were cloned into the vector pCMV-3HA (Clontech, Mountain View, CA, USA). The mature human CCL2 was cloned into pCMV-3HA after removing the N-terminal 23 amino acids of the human CCL2 precursor.

The BFV infectious clone (pBS-BFV-Z1), pCMV-3HA-BEnv, pCE-puro-3Flag-BGag, and pCE-puro-3Flag-Lck-BGag were previously described [12,19]. Mutations were generated by site-directed polymerase chain reaction (PCR) (Toyobo, Osaka, Japan). All mutant plasmids were verified by DNA sequencing (Genewiz, Beijing, China) before use.

Antibodies used for protein analysis were as follows: anti-Flag, anti-HA, anti-Tubulin, and secondary antibodies labeled with horseradish peroxidase (HRP) were purchased from Santa Cruz Biotechnology (Santa Cruz, CA, USA). With reference to the established laboratory protocol for preparing antibodies [37], antibodies against human CCL2 were generated in BALB/c mice using bacterially purified human CCL2 (24–99aa) protein as immunogens. Because human CCL2 (1–99 aa) forms CCL2 (24–99 aa) after being cleaved by signal protein, CCL2 antibody can be used to detect two forms of CCL2.

### 2.3. Quantitative Real-Time Reverse Transcription PCR (qRT-PCR)

The total RNA was extracted using Trizol reagent (Invitrogen Corp, Carlsbad, CA, USA) following the manufacturer’s protocol. The extracted RNA was reverse transcribed into cDNA, and the cDNA was used as a template. qPCR was performed on the StepOnePlus Real-Time PCR System (Applied Biosystems, Foster City, CA, USA) using FastStart Universal SYBR Green PCR Master Mix (Roche, Basel, Switzerland). GAPDH was used as an internal control. All data were measured in triplicates and calculated using the 2^−∆∆CT^ method. The primers for human CCL2 were forward: 5′-CAGCCAGATGCAATCAATGCC-3′ and reverse: 5′-GGAATCCTGAACCCACTTCT-3′.

### 2.4. Generation of Stably Transduced Knockdown Cell Lines

The CCL2 knockdown HEK293T cell line was created using a small hairpin RNA (shRNA). A small hairpin RNA (shRNA) targeting CCL2 was designed using shRNA Sequence Designer (Clontech, Mountain View, CA, USA) and cloned into the pSIREN-RetroQ vector (Clontech, Mountain View, CA, USA). The target sequences for the CCL2 shRNA and control shRNA construct were as follows: 5′-CCCAGTCACCTGCTGTTATAA-3′ and 5′-GAAGTAAGCGATATACATA-3′.

HEK293T cells were transfected with 1 μg pMLV-Gag-Pol, 0.5 μg pVSV-G and 1 μg pSIREN-RetroQ construct. At 48 h after transfection, the supernatants were harvested to infect HEK293T cells to knock down CCL2. The knockdown efficiency of CCL2 was assessed using Western blotting or qPCR.

### 2.5. Purification of BFV Subviral Particles/Virus-like Particles (SVPs/VLPs)

At 48 h after transfection, the cell culture supernatants containing BFV SVPs (including BEnv-only and BGag-BEnv SVPs) and BFV VLPs (Lck-BGag VLPs) were filtered through a 0.45 µm filter, were then added to the centrifuge tube, and then were ultracentrifuged at 12,000× *g* for 3 h at 4 °C. The invisible pellet was resuspended in 40 µL of 1× loading buffer containing 2% SDS and was detected by Western blotting.

### 2.6. Immunofluorescent Assay

HeLa cells were seeded onto 22 mm diameter coverslips and transfected with the corresponding plasmids. At 48 h after transfection, the cells were washed two times with phosphate-buffered saline (PBS), treated with fixative (PBS containing 4% formaldehyde) at room temperature for 10 min and permeabilized with 0.1% Triton X-100 for 10 min at room temperature. Subsequently, 500 µL of blocking buffer (5% nonfat milk and 5% BSA in PBS) was added to each well and blocked for 2 h at room temperature or overnight at 4 °C. The cells were then incubated with anti-Flag or anti-HA antibodies (1:200 dilution) for 2 h at room temperature or overnight at 4 °C. After they had been washed three times with PBS containing 0.1% Tween 20^®^ (PBS-T), the cells were incubated for 40 min with fluorochrome-conjugated secondary antibodies in the dark. Finally, the cells were stained with 4′,6-diamidino-2-phenylindole (DAPI) (diluted 1:2000 in PBS) for 10 min in the dark. The coverslips were fixed with 90% glycerol-PBS and air-dried at room temperature in the dark. The images were captured by a confocal fluorescence microscope (Leica TCS SP5, Wetzlar, Germany).

### 2.7. Western Blotting

Cells were harvested and lysed with lysis buffer (50 mM Tris, 50 mM NaCl, 3% Glycerol, 1% NP-40, 2 mM EDTA) on ice for 40 min, and then were terminated with protein loading buffer containing 2% SDS. Cell lysates or immunoprecipitated materials were boiled at 100 °C for 20 min and then subjected to SDS-PAGE (10 to 15% polyacrylamide). The proteins were transferred to polyvinylidene difluoride (PVDF) membrane (GE Healthcare, Chicago, IL, USA). The blotted PVDF membranes were blocked with PBS containing 5% nonfat milk for 45 min at room temperature, followed by 90 min of primary antibody incubation and 45 min of secondary antibody incubation, respectively. Finally, the substrate for horseradish peroxidase was added to the membrane for 2 min and the immunoreactive proteins were detected by chemiluminescence (Merck Millipore, Darmstadt, Germany).

### 2.8. Co-Immunoprecipitation (Co-IP)

The plasmids required for the experiment were transfected into HEK293T cells, and the cells were harvested after 48 h. Samples were then lysed with immunoprecipitation buffer (50 mM Tris, 150 mM NaCl, 2 mM EDTA, 3% Glycerol, 1% NP-40, EDTA-free protease inhibitor cock-tail tablets) and briefly sonicated. The sonicated samples were centrifuged at 4 °C and 10,000× *g* for 10 min. The supernatant was incubated with antibodies at 4 °C for 3 h, and then incubated with protein A-agarose at 4 °C for 3 h with rotation. The immunoprecipitated components in the pellet were washed 6 times with lysis buffer, and then boiled with 2× loading buffer containing 2% SDS for 20 min at 100 °C. The samples were analyzed using Western blotting.

### 2.9. Statistical Analysis

In the Western blot experiments, the measurement of protein band intensity was obtained by comparing the gray scale measurement with Image J (1.50i) software [38]. To quantify the level of SVPs/VLPs release, the amount of BGag released into the supernatant by SVPs/VLPs was normalized to the amount of intracellular BGag, which was first normalized to the Tubulin control. All data are from the independent experiments in triplicate and are expressed as mean ± standard deviation (SD). The differences between the two groups were compared using the Student’s *t*-test and GraphPad Prism version 8.0 (GraphPad software Inc, San Diego, CA, USA). Differences were considered statistically significant when *p*-values were less than 0.05; * *p* < 0.05, ** *p* < 0.001, and *** *p* < 0.0001 were considered significant, not significant (ns) for *p* > 0.05.

## 3. Results

### 3.1. CCL2 Promotes BFV SVPs Budding

It has been previously reported that CCL2 enhances HIV-1 virus particles release [25]. Since BFV buds in a similar way to HIV-1, we sought to investigate the effects of CCL2 on BFV budding. The bovine Alix sequences cannot be found in NCBI and Alix and Tsg101 in MDBK cells could be detected with antibodies recognizing human ESCRT protein, which suggested that that high similarity of ESCRT proteins between humans and cows [19,39]. BFV has a wide range of phagocytosis, which can be quite easily confirmed by virus isolation in different types of cells (Cf2Th, BoMac, MDBK, KTR, BHK21) [16]. Therefore, to explore the detailed mechanism, we used human ESCRT protein and human cells to complete the whole experiment. To do so, we first examined the effect of CCL2 on the release of BGag-BEnv subviral particles (SVPs). For this purpose, we co-transfected the pCMV-3HA-BEnv plasmid with the pCE-puro-3Flag-BGag plasmid to detect the production of SVPs. Consequently, in the case of co-transfection of pCMV-3HA-BEnv and pCE-puro-3Flag-BGag plasmids, two parts of BEnv protein in the supernatant can be detected with anti-HA: BEnv-only SVPs [40,41] and BGag-BEnv SVPs [42], while anti-flag can only detect BGag bound to BEnv in the supernatant. Therefore, the level of BEnv in the supernatant cannot be regarded as a release level, we used the release of BGag protein in the supernatant as a measure of the release level of BFV SVPs.

**Figure 1:**
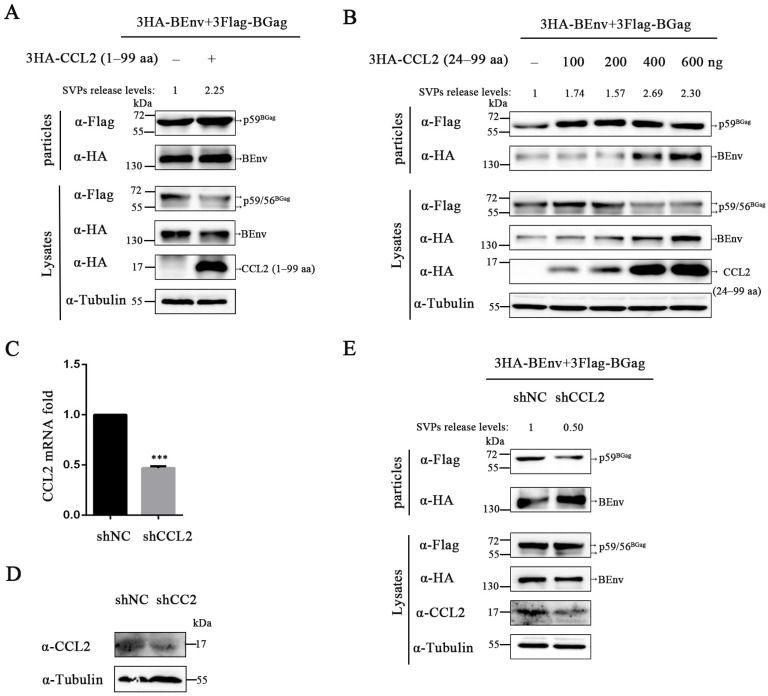
Effects of CCL2 protein on BFV SVPs budding. (**A**,**B**) HEK293T cells (5 × 10^5^) were transfected with pCMV-3HA-BEnv, pCE-puro-3Flag-BGag (BFV Gag protein), the pCMV-3HA, pCMV-3HA-CCL2 (1–99 aa) (CCL2 precursor), or pCMV-3HA-CCL2 (24–99 aa) (CCL2 mature body). Cell culture supernatants were filtered through a 0.45 µm filter and purified by centrifugation. Cell samples are lysed using lysis buffer. The protein levels in cells and supernatants were detected by Western blotting. To further quantify the level of released SVPs, the amount of intracellular BGag was first normalized to the Tubulin control, and then the amount of BGag in SVPs was normalized relative to the amount of intracellular BGag. (**C**,**D**) HEK293T cells (5 × 10^5^) were transduced with lentiviral vectors carrying either a control scrambled shRNA or a CCL2 shRNA. (**C**) The total RNA of cells was extracted and reverse transcribed into cDNA, and the knockdown effect was detected by qPCR. The data shown in the figures are the mean of three independent experiments, and error bars represent the mean; *** *p* < 0.0001. (**D**) CCL2 levels were determined by immunoblotting. (**E**) pCMV-3HA-BEnv and pCE-puro-3Flag-BGag were transfected in control cells and CCL2 knockdown cells. The cell culture supernatants and transfected cells were processed as described in (**A**,**B**). Proteins were also detected and quantified as in (**A**,**B**).

Since CCL2 exists in cells in two forms, the effects of both forms on BFV SVPs budding were investigated separately. As shown in Figure 1A,B and Figure S1A,B, overexpression of CCL2 precursors and mature forms increased the amount of Gag in BFV SVPs.

Next, we investigated the impact of endogenous CCL2 on BFV budding. To determine the knockdown effect specifically targeting CCL2, we examined the mRNA and protein levels of CCL2 in knockdown cell lines (Figure 1C,D). The CCL2 knockdown significantly inhibited the release of BFV SVPs compared to the control (Figure 1E and Figure S1C). These findings suggest that CCL2 plays a crucial role in the budding of BFV SVPs.

### 3.2. CCL2 Eanhances BFV VLPs Budding

**Figure 2:**
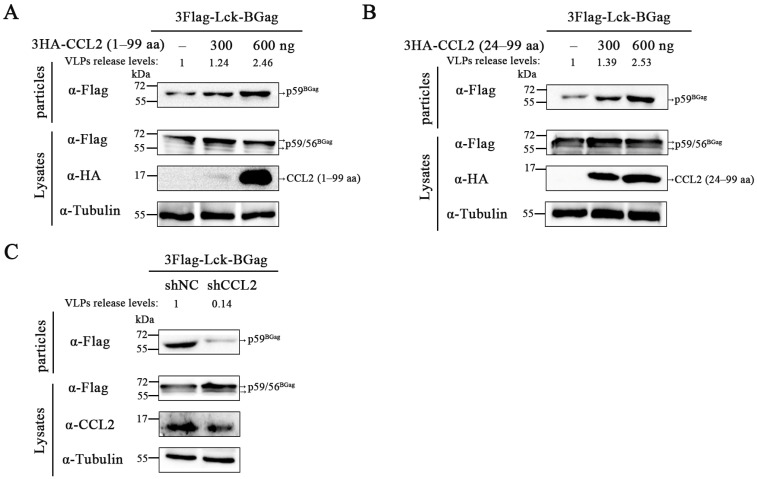
Effects of CCL2 protein on BFV VLPs budding. (**A**,**B**) HEK293T cells were transfected with pCE-puro-3Flag-Lck-BGag, pCMV-3HA-CCL2 (1–99 aa), or pCMV-3HA-CCL2 (24–99 aa). (**C**) pCE-puro-3Flag-Lck-BGag was transfected in control cells and CCL2 knockdown cells. (**A**–**C**) At 48 h post-transfection, samples were collected. The supernatants of the cell culture were filtered through a 0.45 μm filter and purified by ultracentrifugation. Transfected cells were disrupted with lysis buffer. Protein levels in cells and supernatants were measured by Western blotting. To further quantify the level of VLPs release, the amount of intracellular BGag was initially normalized to the Tubulin control, and then the amount of BGag in VLPs was normalized relative to the amount of intracellular BGag.

In order to confirm the effect of CCL2 on BFV budding and exclude the interference of BEnv protein, the study explored the effect of CCL2 on the release of BFV VLPs. To achieve this, we enabled Lck-BGag to be targeted to the plasma membrane and released alone by adding the myristoylation sequence of Lck oncoprotein to the N-terminus of BGag. Subsequently, we transfected pCE-puro-3Flag-Lck-BGag plasmid to detect the production of VLPs. As shown in Figure 2A,B and Figure S2A,B, overexpression of CCL2 precursors and mature forms increased the release of Lck-BGag in the supernatant. To determine the effect of endogenous CCL2 on BFV VLPs release, the level of BGag in the supernatant after knockdown of CCL2 was examined. The results showed that knockdown of CCL2 significantly reduced BGag release (Figure 2C and Figure S2C). Collectively, these findings suggest that CCL2 promoted the release of BFV VLPs independently of BEnv.

### 3.3. CCL2-Responsiveness Is Dependent on the Presence of YGPL Motif in BFV Gag and Alix in the Cell

**Figure 3:**
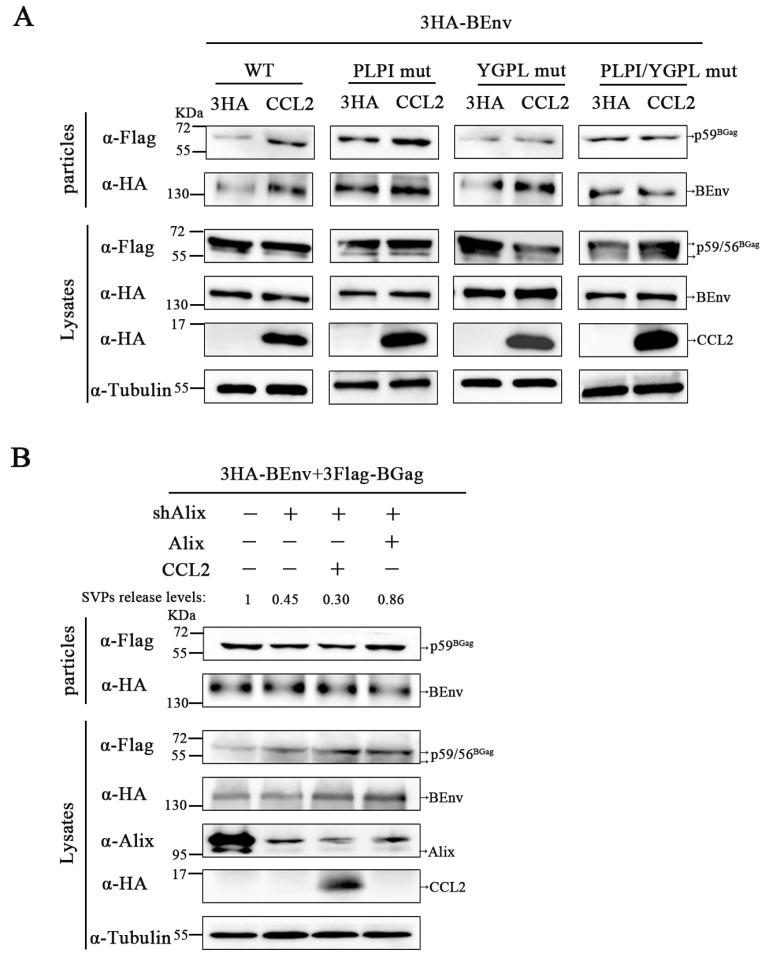
CCL2-mediated changes in virus budding requires the presence of BGag YGPL motif in the virus and ALIX in target cells. (**A**) HEK293T cells were transfected with pCMV-3HA-BEnv, pCE-puro-3Flag-BGag (wild type, L domain mutant PLPI/AAAA, L domain mutant YGPL/AAAA), pCMV-3HA-CCL2 (24–99 aa), or the empty vector. Samples were collected after 30 h of transfection. Cell culture supernatants were filtered through 0.45 µm filters and purified by centrifugation. Cell samples were lysed with lysis buffer. Protein levels in the samples were measured by Western blotting. (**B**) The control cells and Alix knockdown cells were transfected with pCMV-3HA-BEnv, pCE-puro-3Flag-BGag, the empty vector or pCMV-3HA-CCL2 (24–99 aa), and then cultured for 48 h. The cell culture supernatants were filtered through a 0.45 µm filter and purified by ultracentrifugation. Protein levels in cells and supernatants were determined by Western blotting. The levels of released SVPs were quantified according to the method described in the previous data analysis.

Recent studies have demonstrated that promotion of HIV-1 release by CCL2 is dependent on the LYPX motif contained in the L domain of Gag-p6 [25]. In previous laboratory studies, the PLPI and YGPL motifs in BGag were found to contain L domain activity for BFV particles budding. To determine the role of the L domain motifs of BGag in CCL2-mediated promotion of BFV budding, pCMV-3HA-BEnv and plasmids expressing different BGag proteins were co-transfected into HEK293T cells. As shown in Figure 3A, when the YGPL motif of BGag was mutated, overexpression of CCL2 no longer promoted the release of BFV SVPs, whereas when the YGPL motif of BGag was present, CCL2 increased the release of BGag in the supernatant. In summary, the YGPL mutation in BFV eliminated CCL2-responsiveness and the effects of CCL2 were mediated by the YGPL motif, but not by the PLPI motif.

As previous studies have found that the YGPL motif can bind Alix, an auxiliary protein of ESCRT-III, it was hypothesized that the effects of CCL2 are mediated by Alix. To test this hypothesis, the role of Alix in CCL2-responsiveness was examined by knocking down Alix. Co-transfection of pCMV-3HA-BEnv and pCE-puro-3Flag-BGag plasmids into Alix knockdown HEK293T cells resulted in reduced virus budding (SVPs release levels were 45% compared to control) and was not further increased by overexpression of CCL2 (Figure 3B). These results confirm that responsiveness to CCL2 requires YGPL motif in the virus and the presence of Alix in the cell.

### 3.4. CCL2 Facilitates BGag-Alix Binding and Alix Mediates the Association of BGag with CCL2

In terms of the contribution of BFV Gag (BGag) binding to Alix toward virions budding and release, and based on the fact that CCL2 responsiveness is dependent on the presence of Alix (Figure 3B), we hypothesized that CCL2 may mediate its effect by enhancing the interaction between BGag and Alix.

**Figure 4:**
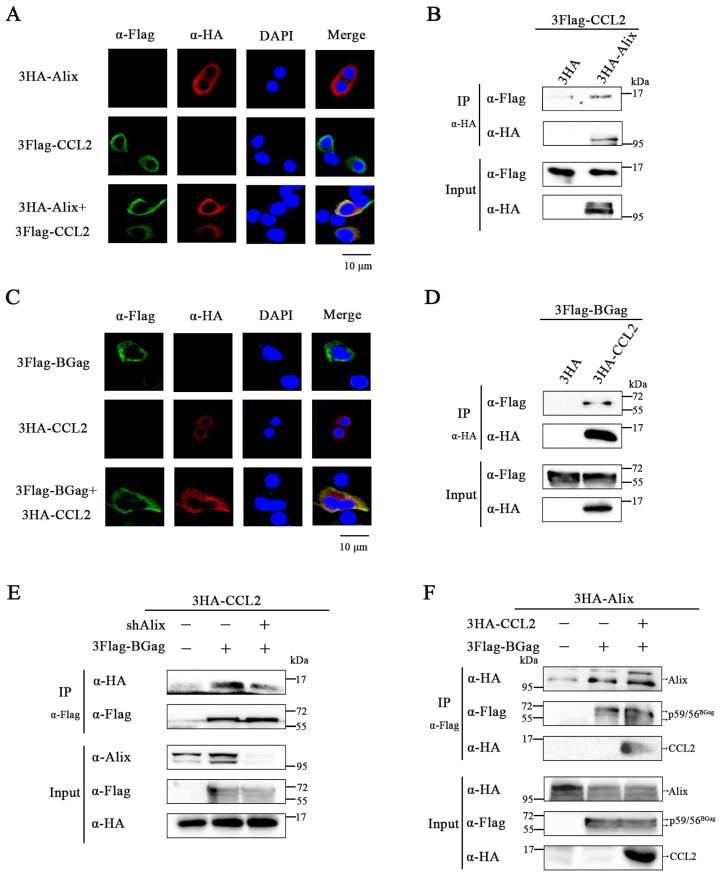
CCL2 enhances the interaction between Bgag and Alix and Alix links Bgag to CCL2. (**A**) HeLa cells were co-transfected with pCMV-3HA-Alix, pCE-puro-3Flag-CCL2 (24–99 aa), or co-transfected with their combinations, respectively, for 48 h. Indirect immunofluorescence assay (IFA) was used to localize CCL2 (using rabbit anti-HA antibody and tetramethyl rhodamine isocyanate (TRITC)-conjugated goat anti-rabbit secondary antibody) and Alix (using mouse anti-Flag antibody and isothiocyanate (FITC)-conjugated goat anti-mouse secondary antibody). The cell nuclei were stained with 4′,6-diamidino-2-phenylindole (DAPI) staining. Scale bar = 10 μm. (**B**) HEK293T cells (4 × 10^6^) were transfected with pCE-puro-3Flag-CCL2, the empty vector or pCMV-3HA-Alix. Co-IP with HA antibody was performed after 48 h of transfection. Samples of cell lysates and immunoprecipitates were measured by Western blotting. (**C**) HeLa cells were co-transfected with pCMV-3HA-CCL2 (24–99 aa), pCE-puro-3Flag-BGag or co-transfected with their combinations, respectively, for 48 h. Cells were subjected to immunofluorescence staining using an anti-Flag antibody (FITC) or anti-HA antibody (TRITC), and the cell nuclei were stained with DAPI. Scale bar = 10 µm. (**D**) pCE-puro-3Flag-BGag, pCMV-3HA-CCL2 (24–99 aa) or the empty vector were transfected into HEK293T cells (4 × 10^6^). Co-IP with HA antibody was performed at day 2 post-transfection. Detection of protein in cell lysates and immunoprecipitates was performed using HA and Flag antibodies. (**E**) pCMV-3HA-CCL2, the empty vector or pCE-puro-3Flag-BGag were transfected in the control cells and Alix knockdown cells (4 × 10^6^). Co-IP with Flag antibody was performed after 48 h of transfection. Western blotting of samples from cell lysates and immunoprecipitates using Alix, anti-Flag, and anti-HA antibodies. (**F**) HEK293T cells (4 × 10^6^) were transfected with pCMV-3HA-Alix, pCE-puro-3Flag-BGag or pCMV-3HA-CCL2 (24–99 aa). Co-IP with Flag antibody was performed after 48 h of transfection. Cell lysates and immunoprecipitates were subjected to Western blotting in which anti-Flag and anti-HA antibodies were used.

To investigate this hypothesis, we first examined the relationship between CCL2 and Alix. We examined the subcellular localization of CCL2 and Alix using immunofluorescence detection. As shown in Figure 4A, the CCL2 and Alix were co-localized in the cytoplasm. We further detected a distinct interaction between Alix and CCL2 by Co-IP (Figure 4B and Figure S3A). These results suggested that CCL2 interacts with Alix. Subsequently, we explored whether there is just an interaction between CCL2 and BGag. Immunofluorescence staining showed that CCL2 co-localized with BGag (Figure 4C). Co-IP was further confirmed the association between CCL2 and BGag (Figure 4D and Figure S3B). These data suggested an interaction between CCL2 and BGag.

To determine whether Alix mediates the interaction between BGag and CCL2, we performed Co-IP in control HEK293T cells and Alix knockdown HEK293T cells. We observed a distinct weakening of the interaction between BGag and CCL2 after knockdown of Alix (Figure 4E and Figure S3C). These results suggest that Alix acts as a bridge between BGag and CCL2.

Based on these findings, we verified our hypothesis and showed that Alix interacts with BGag (Figure 4F), consistent with previous studies. We also found that the presence of CCL2 enhanced the ability of BGag to bind Alix, which was confirmed by an increase in the amount of Alix bound to BGag in the presence of CCL2 compared to its absence. Additionally, we detected the presence of CCL2 in the precipitates of BGag and Alix, suggesting that CCL2, BGag, and Alix may be related to each other to some extent (Figure 4B,D).

Overall, our results indicate that CCL2 promotes BFV budding by enhancing the ability of BGag to bind Alix, and that Alix acts as a bridge between BGag and CCL2.

### 3.5. Bovine CCL2 Facilitates BFV Budding

**Figure 5:**
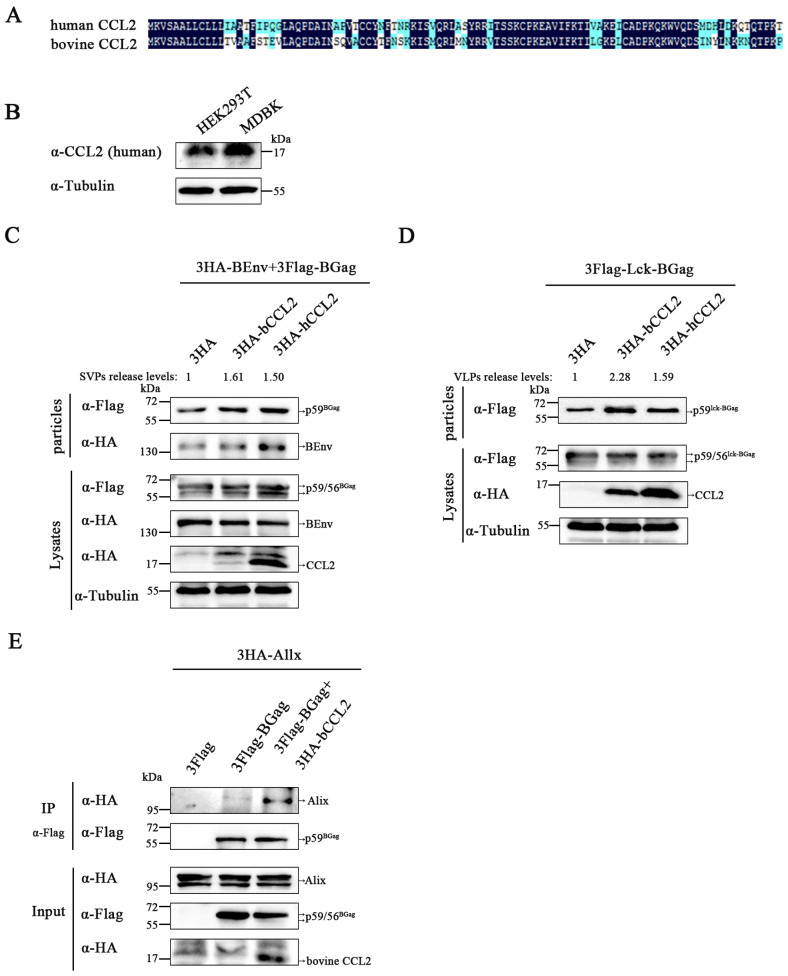
Effects of bovine CCL2 on particle budding. (**A**) The full-length amino acid sequences of bovine CCL2 and human CCL2 were analyzed using DNAMAN (6.0.3.99) software. (**B**) Equal amounts of cells (HEK293T, MDBK) were harvested and analyzed for endogenous CCL2 protein levels using Western blotting. (**C**) HEK293T cells were transfected with pCMV-3HA-BEnv, pCE-puro-3Flag-BGag, pCMV-3HA-bovine-CCL2 (3HA-bCCL2) or pCMV-3HA-human-CCL2 (3HA-hCCL2) or the empty vector. (**D**) pCE-puro-3Flag-Lck-BGag, pCMV-3HA-bCCL2 or pCMV-3HA-hCCL2 or the empty vector were transfected into HEK293T cells. (**C**,**D**) At 48 h post-transfection, cell culture supernatants were filtered through a 0.45 µm filter and purified by centrifugation. Cell samples are lysed using lysis buffer. The protein levels in cells and supernatants were detected by Western blotting. To further quantify the level of released SVPs/VLPs, the amount of intracellular BGag was first normalized to Tubulin control, and then the amount of BGag in SVPs/VLPs was normalized relative to the amount of intracellular BGag. (**E**) HEK293T (4 × 10^6^) cells co-transfected with pCMV-3HA-Alix and pCE-puro-3Flag-BGag or pCMV-3HA-bCCL2 were immunoprecipitated with Flag antibody. Mouse anti-HA antibody was used to detect Alix, CCL2 and mouse anti-Flag antibody was used to detect BGag protein.

Previous studies have suggested that human CCL2 (hCCL2) is essential in BFV budding, and in order to validate its effect, experiments were performed with bovine CCL2 (bCCL2), which is a natural host chemokine of BFV. Compared to human CCL2, bovine CCL2 has no precursor and mature forms [43] but shows a 71.72% amino acid sequence similarity (Figure 5A). The similarity of the CCL2 proteins was further confirmed by detecting bovine CCL2 using antibodies recognizing human CCL2 in MDBK cell (Figure 5B). The experiments showed that overexpression of bovine CCL2 increased the amount of BGag in BFV SVPs/VLPs, resulting in a more pronounced effect on BFV budding compared to human CCL2 (Figure 5C,D). Furthermore, Co-IP experiments confirmed that bovine CCL2 enhances the interaction between BGag and Alix, which is consistent with the previous findings on human CCL2 (Figure 5F). In conclusion, these results suggest that bovine CCL2 promotes BFV budding by enhancing the interaction between BGag and Alix and has a more potent effect than human CCL2.

## 4. Discussion

CCL2 binds to its receptor CCR2, which regulates cell migration and plays a crucial role in inflammation. Recent studies have shown that CCL2 also has certain effects on viral particle release and replication [32,33]. In the present study, we found that CCL2 plays an important role in BFV budding in HEK293T cells. We examined the effects of increased CCL2 expression and knockdown of CCL2 expression on BFV budding. In both cases, we noted a strong positive correlation between the CCL2 expression level and BFV budding, suggesting that CCL2 promotes BFV particle release. We also identified that CCL2 enhances BGag-Alix binding, allowing BFV to recruit more ESCRT components and facilitating the release of viral particles.

Human CCL2 is very similar to bovine CCL2, and we demonstrated that both proteins promoted BFV budding, and that homologous CCL2 promoted the virus more significantly. Additionally, the sequence of bovine CCL2 and mammalian CCL2 is highly consistent [43]. This suggested that CCL2 from different species might promote BFV budding. CCL2 affects viral release by increasing the level of ALIX-BGag interaction; however, there was no difference in the enhancing the ALIX-BGag interaction of hCCL2 or bCCL2 (Figure 4F and Figure 5E). This type of species-independence is distinct from some host proteins that affect viral replication, such as SGK-1 (encoding serum/glucocorticoid regulated kinase 1), hSGK-1, and bSGK-1 which all inhibit PFV replication. In contrast, heterologous SGK-1 inhibits PFV more effectively [44], or for instance Myxovirus resistance 2 (Mx2), which is present in multiple primate species that have the ability to potently inhibit HIV-1, whereas the selected non-primate orthologs have no such activity [45].

Typically, ESCRT complexes are responsible for vesicular trafficking within the cell, which are co-opted by the retroviral Gag polyprotein to assist in viral particle assembly and release of infectious virions. Several viruses are known to use their L domain to recruit ESCRT complexes for viral budding, including HIV-1, equine infectious anemia virus (EIAV), and murine leukemia virus (MuLV) [46]. Among them, the Gag P6 structural protein interacts with Tsg101 and Alix to promote HIV-1 release. Additionally, CCL2 is also involved in the ESCRT-mediated release pathway and promotes increased HIV-1 viral release and improved replication fitness [25]. In the present study, we suggest that the ability of CCL2 to promote BFV budding is primarily dependent on the enhanced interaction of BGag with Alix. Further, the level of CCL2 is also regulated by viral infection, such as severe acute respiratory syndrome coronavirus [47] and human cytomegalovirus [48]. Here, we demonstrated that BFV infection of HEK293T induces CCL2 levels (Figure S4). These viruses with the L domain mentioned above are known to induce CCL2, suggesting that viral budding using CCL2-enhanced ALIX-Gag interaction may be a feature shared by different viruses.

Our data suggests a different means by which the level of CCL2 affects Alix’s interaction with BGag, as a result of affecting the release of the virus. This is consistent with the promotion of HIV-1B release by CCL2, which enhances the binding of Alix and P6 through CCL2 [25]. In the HIV-1 B branch, CCL2 facilitates its budding dependent on the classic L domain LYPX as well as the presence of Alix in cells. In our experiment, we observed that the function of CCL2 to promote BFV budding was lost when the atypical L domain YGPL motif was not present (Figure 3A). And this promotion is independent of the L domain PLPI motif of BFV. These results suggest that the budding of viruses containing non-classical L domain motif and interacting with Alix may be regulated by CCL2. Interestingly, one finding from our data is that there is a certain association between Alix, BGag, and CCL2, while Alix mediates the interaction between CCL2 and BGag. Furthermore, this is the first report of the relationship between CCL2, BGag, and Alix in this way.

In summary, we found that CCL2 plays a vital catalytic role in BFV budding. CCL2 enhances the interaction between BGag and Alix, which recruits more ESCRT components to mediate viral budding. These results suggest that CCL2 might promote the budding of some retroviruses without the classical L domain motif in their structural proteins. Hence, our findings not only shed novel insights into the recruitment of more ESCRT complexes by viral proteins containing atypical L domain motif, they also further our understanding of CCL2 function and provide a new clue to the release of retroviruses with atypical L domain motif.

## Data Availability

The data presented in this study are available on request from the corresponding author.

## Supplementary Materials

The following supporting information can be downloaded at: https://www.mdpi.com/article/10.3390/v15091867/s1, Figure S1: (A–C) Quantification of BFV SVPs budding. Mean values and standard deviation of particle-associated BGag protein corrected for intracellular expression levels are shown. To quantify the SVPs release levels, the amount of BGag in SVPs was normalized against the amount of intracellular BGag, which were first normalized against the Tubulin loading control. The data shown in figures are the mean of three independent experiments, compared with the control: * *p* < 0.05, ** *p* < 0.01; Figure S2: (A–C) Quantification of BFV VLPs budding. Mean values and standard deviation of particle-associated BGag protein corrected for intracellular expression levels are shown. To quantify the VLPs release levels, the amount of BGag in VLPs was normalized against the amount of intracellular BGag, which were first normalized against the Tubulin loading control. The data shown in figures are the mean of three independent experiments, compared with the control: ns for *p* > 0.05, * *p* < 0.05, ** *p* < 0.01; Figure S3: (A) Immunoprecipitation with Flag antibody of HEK293T (4 × 10^6^) cells cotransfected with eukaryotic expression plasmids encoding pCMV-3HA-Alix and pCE-puro-3Flag-CCL2 (24–99 aa). (B) Immunoprecipitation with Flag antibody of HEK293T (4 × 10^6^) cells cotransfected with eukaryotic expression plasmids encoding pCMV-3HA-CCL2 (24–99 aa) and pCE-puro-3Flag-BGag. (C) Quantification of the extent of BGag interaction with CCL2. Means and standard deviations are shown for CCL2 protein in immunoprecipitates. To quantify the extent of BGag-CCL2 interaction after knockdown of Alix, the amount of CCL2 in the immunoprecipitates was normalized to the amount of BGag in the immunoprecipitates. Data shown in the graphs are the mean of three independent experiments, compared with the control: * *p* < 0.05; Figure S4: (A,B) HEK293T cells were transfected with an empty vector and pBS-BFV-Z1. (A) 48 h after transfection, samples were harvested and lysed. Detected by Western blotting and indicated antibodies. (B) Samples were collected 48 h after transfection. The total RNA of the cells was extracted and reverse transcribed into cDNA. Data shown in figures are the mean of two or three independent experiments, and error bars represent the mean; *** *p* < 0.0001.
