# Supplementary material for: Effects of Chemokine Ligand 2 on Budding of Bovine Foamy Virus
Source: Viruses. 2023 Sep 1;15(9):1867. doi: 10.3390/v15091867 (PMC10536199; doi:10.3390/v15091867)
Supplement: Supplementary file 1 [file viruses-15-01867-s001.zip › viruses-2442596-supplementary.pdf]

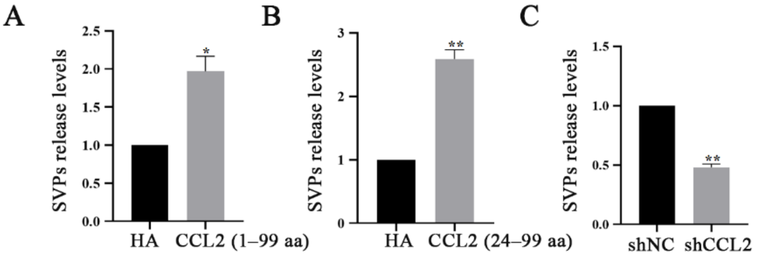

Supplementary Figure S1. (A–C) Quantification of BFV SVPs budding. Mean values and standard deviation of particle-associated BGag protein corrected for intracellular expression levels are shown. To quantify the SVPs release levels, the amount of BGag in SVPs was normalized against the amount of intracellular BGag, which were first normalized against the Tubulin loading control. The data shown in figures are the mean of three independent experiments, compared with the control: * $p < 0.05$, ** $p < 0.01$.

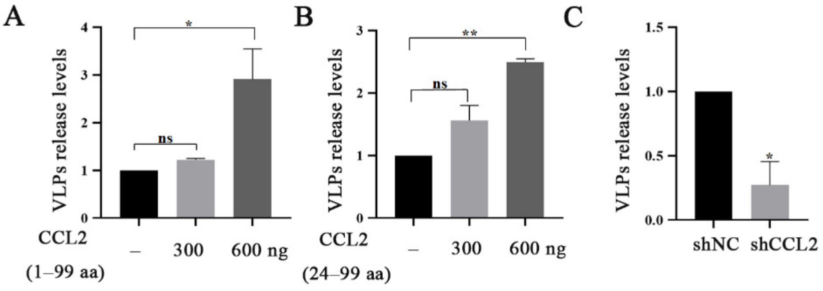

Supplementary Figure S2. (A–C) Quantification of BFV VLPs budding. Mean values and standard deviation of particle-associated BGag protein corrected for intracellular expression levels are shown. To quantify the VLPs release levels, the amount of BGag in VLPs was normalized against the amount of intracellular BGag, which were first normalized against the Tubulin loading control. The data shown in figures are the mean of three independent experiments, compared with the control: ns for $p > 0.05$, * $p < 0.05$, ** $p < 0.01$.

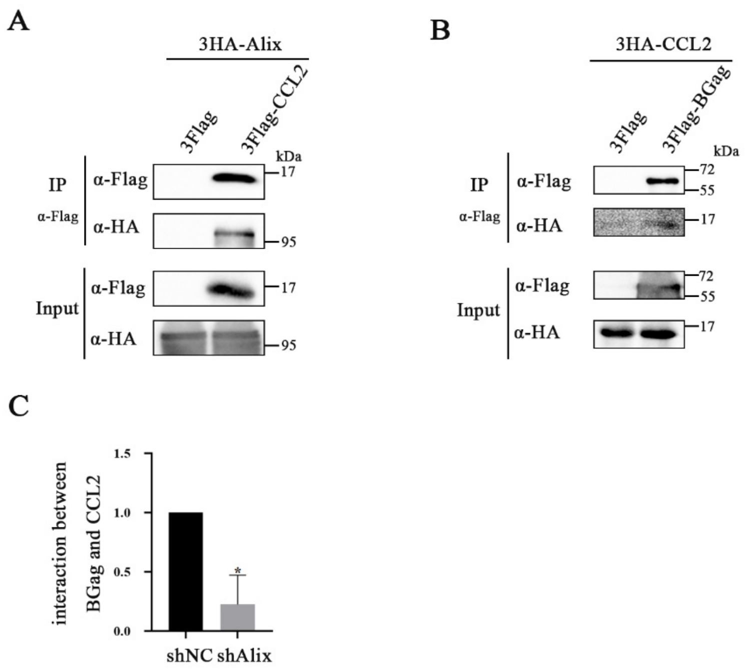

Supplementary Figure S3. (A) Immunoprecipitation with Flag antibody of HEK293T (4×10^6) cells cotransfected with eukaryotic expression plasmids encoding pCMV-3HA-Alix and pCE-puro-3Flag-CCL2 (24–99 aa). (B) Immunoprecipitation with Flag antibody of HEK293T (4×10^6) cells cotransfected with eukaryotic expression plasmids encoding pCMV-3HA-CCL2 (24–99 aa) and pCE-puro-3Flag-BGag. (C) Quantification of the extent of BGag interaction with CCL2. Means and standard deviations are shown for CCL2 protein in immunoprecipitates. To quantify the extent of BGag-CCL2 interaction after knockdown of Alix, the amount of CCL2 in the immunoprecipitates was normalized to the amount of BGag in the immunoprecipitates. Data shown in the graphs are the mean of three independent experiments, compared with the control: *$p < 0.05$.

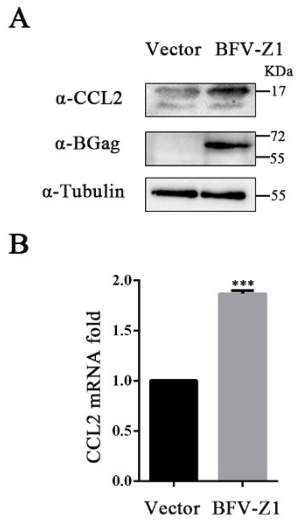

Supplementary Figure S4. (A, B) HEK293T cells were transfected with an empty vector and pBS-BFV-Z1. (A) 48 h after transfection, samples were harvested and lysed. Detected by Western blotting and indicated antibodies. (B) Samples were collected 48 h after transfection. The total RNA of the cells was extracted and reverse transcribed into cDNA. Data shown in figures are the mean of two or three independent experiments, and error bars represent the mean; ***$p < 0.0001$.
